# Effects of Inositol Supplementation in Sperm Extender on the Quality of Cryopreserved Mesopotamian Catfish (*Silurus triostegus*, H. 1843) Sperm

**DOI:** 10.3390/ani11113029

**Published:** 2021-10-21

**Authors:** Zafer Doğu, Erdinç Şahinöz, Faruk Aral, İsmail Koyuncu, Özgür Yüksekdağ

**Affiliations:** 1 Department of Fisheries and Aquaculture, Bozova Vocational High School, Harran University, Bozova 63850, Turkey; e_sahinoz@harran.edu.tr; 2 Çarşı PTT PK 138, Konya 42010, Turkey; faral6@hotmail.com; 3 Department of Medical Biochemistry, Faculty of Medicine, Harran University, Sanliurfa 63050, Turkey; ismailkoyuncu1@gmail.com (İ.K.); ozguryuksekdag47@gmail.com (Ö.Y.)

**Keywords:** apoptosis, cryopreservation, DNA damage, oxidative stress, *Silurus triostegus*, sperm

## Abstract

**Simple Summary:**

This study was based on the determination of post-thaw semen quality, oxidant–antioxidant status and DNA-damage status of frozen Mesopotamian catfish semen. To this end, the sperm was frozen in diluent containing different inositol concentrations (5, 10, 20 and 40 mg). Increasing levels of inositol linearly increased the spermatozoa motility rate and duration significantly (*p* < 0.05). High doses of inositol increased antioxidants and decreased oxidants. In addition, lower intracellular DNA damage and percentage of apoptotic spermatozoa occurred in high doses of inositol (*p* < 0.05).

**Abstract:**

In this study, the effects of supplemented inositol on sperm extenders were examined on the spermatozoa motility rate and duration, total antioxidant and oxidant status, apoptotic spermatozoa and DNA damage, during the sperm post-thaw process of Mesopotamian Catfish (*Silurus triostegus*, H. 1843). The semen was frozen in diluents containing different inositol concentrations (5, 10, 20 and 40 mg). Increasing levels of inositol linearly improved the spermatozoa motility rate and duration significantly (*p* < 0.05). MDA and TOS were linearly decreased, however, TAS and GSH linearly increased (*p* < 0.05). The increasing inositol levels resulted in a linear and quadratic decrease in DNA damage in the comet assay, 8-hydroxydeoxyguanosine and the determined percentage of apoptotic spermatozoa (*p* < 0.05). These results suggest that there are many positive effects of the use of supplemental inositol on enhancing sperm cryopreservation efficiency in *Silurus triostegus*.

## 1. Introduction

*Silurus triostegus* (H. 1843) (Mesopotamian catfish) live only in the Euphrates–Tigris basins [1,2,3,4]. Although there are many studies on the biological properties of *Silurus triostegus*, which are abundantly consumed by the local people, there are few studies on its semen conservation in order to breed the species and protect its genetic resources [1,3,5,6,7].

Sperm freezing protocols continue to be developed to improve the fertilization quality of male fish, as well as humans and some animals. In addition, sperm freezing can be effective in protecting population dynamics against possible risks due to increased stress factors in the natural environments of living organisms, and it is an effective tool for protecting genetic resources [8,9,10]. In addition, it allows the more effective use of gamete control methods, such as hybridization in aquaculture, and studies on subjects, such as refreezing thawed sperm [10,11,12].

In order to develop the optimum sperm freezing medium, different factors such as diluents, cryoprotectants, freezing rates, equilibration and freezing and thawing methods need to be examined separately [10,13,14].

Myo-inositol (MI) is a sugar-like nutrient that is involved in many intracellular mechanisms [15]. High concentrations of MI in the seminal plasma of many animal species contribute to the quality of sperm in the gonads [16,17,18]. Due to its vitamin-like properties, inositol has mostly been tested as a feed supplement for fish [19,20,21,22]. It has been determined that adding antioxidants to the semen extender of some animals has a positive effect on sperm motility [23,24]. It has been reported that inositol affects the osmotic pressure of the seminal fluid and that inositol added to the semen extender increases cytosolic and internal mitochondrial Ca^++^ [25,26,27]. Inositol with antioxidant properties can reduce MDA formation by counteracting intracellular ROS and causing increased sperm viability and motility after thawing [28]. It has been reported that MI reduces DNA damage in ram, bull, dog and human sperm DNA after the freeze–thaw process [26,29]. However, no information has been found about MI effect on *Silurus triostegus* sperm.

After the thawing it has been reported that mitochondrial damage, lipid peroxidation and DNA damage occurs with the increase in cytoplasmic ROS and that sperm motility therefore decreases [30,31,32,33,34]. Although numerous studies have measured these parameters individually, none have measured all parameters in the same sperm population and under the same conditions. In addition, many of these data are not available for *Silurus triostegus* sperm and the data that are available are limited. Since all species have some differences in sperm characteristics, the sperm respond differently to exogenous substances they encounter and to the freeze–thaw process [35,36,37].

Although it is generally accepted that sperm motility decreases with cryopreservation, the reason for this is not yet clear. Park et al. [38] found that ROS production increased during the freeze–thaw process of semen; when ROS increased, it led to lipid peroxidation in the sperm [39,40,41,42]. The lipid peroxidation product malondialdehyde (MDA) causes DNA damage [40]. High levels ROS cause the degradation of nucleic acids and lipids, finally leading to cell death [38,43,44]. ROS is also a known inducer of apoptosis in mature sperm [45,46]. Mitochondria play an important role in ROS generation [47].

In order for the sperm to remain fertile its motility should be high, and DNA damage and apoptosis should be low during the freeze–thaw process. It has been reported that oxidants, which cause these disorders in sperm, should be kept low in semen diluents or supplemented with antioxidants [32]. Antioxidants in fresh semen originate from the seminal plasma [48], and it is accepted that they protect semen quality and the DNA structure against ROS formed in various forms, especially via sperm mitochondria [34].

Maintaining sperm cell integrity is essential for both motility and fertility. Decreased motility after freezing and thawing is associated with damage to the sperm cell [30,34]. Therefore, it was not possible to determine whether the decrease in sperm motility could be explained entirely by loss of function and DNA damage due to oxidative damage. In addition, antioxidants added to semen extenders differ in their effects on motility, oxidative damage, antioxidant levels, DNA damage and apoptosis.

Supplementing the extender with antioxidants during cryopreservation improved semen quality after thawing [49]. It has been reported that inositol, melatonin and quercetin caused a significant improvement in post-thaw sperm motility and DNA integrity [38,43,44,50,51,52,53]. In addition, it has been determined that inositol increases mitochondrial calcium levels, improves sperm mitochondrial function with an antioxidative effect and prevents apoptosis [32]. Governin et al. [54] demonstrated the ability to prevent oxidation damage in DNA with 20 mg/mL MI, with a significant decrease in the level of 8-OHdG in sperm. It has been shown in different studies that the antioxidant properties of inositol on sperm quality, oxidant and antioxidant levels and DNA damage, when added before cryo-storage, differ according to the type of animal used. In contrast, the effects of inositol supplementation to the semen extender before freezing on *Silurus triostegus* sperm are unknown.

Cryopreservation plays a critical role in aquaculture, such as for the preservation of sperm, the protection of endangered genetic resources and the breeding of alternative species. Studies show that sperm conservation is very important for female and male catfish species that have synchronization problems during breeding periods, as well as for the protection of the genetic materials of endemic species [55]. From this point of view, an extender was tested for the preservation of the *Silurus triostegus* semen that is endemic to the Euphrates–Tigris basin.

The main purpose of sperm freezing is to obtain highly motile spermatozoa after thawing by maintaining the number of live spermatozoa. The effects of supplementing the semen extender with inositol on the motility rate and duration, total antioxidant and oxidant status, DNA damage and apoptosis of spermatozoa were examined after the thawing process.

## 2. Materials and Methods

### 2.1. Study Area and Semen Collection

This study was carried out in the research laboratory of the Biochemistry Department of the Harran University Faculty of Medicine. Preliminary examinations of *Silurus triostegus* semen samples were carried out in the laboratories of the Fisheries Department of Harran University Bozova Vocational School.

In the study, five- and six-year-old catfish were used (*n* = 7), that were obtained from fishermen in Atatürk Dam Lake, and age estimates were obtained from the vertebrae. Color, jaw teeth and pectoral fins were assessed to verify the species. The size of captive *Silurus triostegus* were between 1900.00 and 2400.00 g (mean 2148.75 ± 164.78 g) in body weight and between 60.00 and 72.00 cm (mean 66.87 ± 3.56 cm) in total length.

The urogenital papilla of the caught fish was carefully dried, and contact with urea or feces was prevented. Then, semen was collected in 5 mL glass tubes by applying abdominal massage, and the tubes were immediately placed in a Styrofoam container with ice (4 ± 2 °C) and transferred to the laboratory.

### 2.2. Chemicals

Myo-inositol, DMSO and other chemicals were purchased from Sigma-Aldrich (St. Louis, MO, USA).

### 2.3. Sperm Evaluation

#### Sperm Motility, Duration, and Concentration

For the determination of spermatological characteristics, 5 µL of fresh and thawed semen was mixed with 25 µL of activation solution (0.29% NaCl) on the slide, and the motility rate and duration were evaluated under a light microscope (400× magnification) after the coverslip was closed [56]. The sperm were diluted in a counting medium (3.2% sodium citrate with 1% formaldehyde) and calculated by counting on a Thoma slide using the hemocytometric method [57].

After the examination, samples were excluded from the study if they had an abnormal appearance or macroscopic pathological disorders, did not produce semen, had spermatozoa motility below 80%, did not have motile sperm or did not have a semen concentration of 9.00 × 10^9^/mL [58,59]. Approximately 1.5 mL of semen was taken from each sample and centrifuged at 2000× *g* for 30 min to measure seminal plasma osmotic pressure and pH level. Subsequently, all suitable semen samples were mixed to avoid individual variation, and a semen pool was created.

### 2.4. Cryopreservation Protocol

Glucose (0.3 M) was used as diluent and 10% dimethyl sulfoxide (DMSO) was added. MI was not added in the control group, and 5, 10, 20 and 40 mg/mL of MI was added to experimental group extenders. The dilution in all groups was 1/3 (one part sperm to three parts diluent).

The osmolality of sperm (approximately 500 µL) from each fish was cryoscopically measured at room temperature (Osmometer 3250, Advanced Instruments Inc., Norwood, MA, USA).

After dilution, the samples were immediately drawn into 0.25 mL straws (IMV, France). In order to freeze the sperm it was kept in liquid nitrogen vapor, 5–7 cm above the surface of the liquid nitrogen, in a Styrofoam container for 12 min at approximately −110 °C, then stored in liquid nitrogen (−196 °C; MVE Millennium XC 20, Chart Industries, New Prague, MN, USA). After being stored in liquid nitrogen for 2 weeks, the frozen semen was thawed and analyzed. Semen samples frozen in extenders containing inositol and control group extenders were thawed by keeping them at 40 °C for 8 s [60]. The application was repeated three times for the *Silurus triostegus* sperm, and seven straws were frozen for each group.

### 2.5. Preparation of Sperm Samples for Biochemical Analysis

In this study, equal amounts of sperm samples were taken from all groups. Sperm samples were diluted 1/10 with PBS, homogenized with a homogenizer (Tissue Lyser LT, Qiagen, Hilden, Germany) and centrifuged to obtain supernatants.

#### 2.5.1. Oxidative Stress Analysis

The effects of extenders containing inositol on the oxidative stress indices of semen were evaluated by examining the total antioxidant status (TAS), total oxidant status (TOS) and glutathione (GSH) and malondialdehyde (MDA) levels.

GSH levels were evaluated via a reaction with OPA (1 mg/mL o-phthaldialdehyde in methanol), following the adapted technique of Kand’ar and Hajkova [61], with GSH used as a standard. GSH samples were evaluated using a microplate reader (SpectraMax, M5, San Jose, CA, USA), with stimulation at 345 nm and emission at 425 nm. The results were expressed as nmol/mL and nmol/g in the sperm.

MDA levels in the sperm were determined by following a technique defined by Ohkawa et al. [62]. ELISA plates were read through a microplate reader (SpectraMax M5, SpectraMax M5, San Jose, CA, USA) at 532 nm. The results were obtained as nmol/mL and nmol/g in the sperm.

#### 2.5.2. Measurement of Oxidative Stress Status

TAS and TOS were determined in sperm homogenates by using commercially available kits (Rel Assay^®^, Diagnostics kits, Mega Tıp, Gaziantep, Turkey) with an autoanalyzer (Cobas Integra 800, Roche Diagnostics, Indianapolis, IA, USA). TAS and TOS results were shown in mmol H_2_O_2_ equivalent/L and mmol Trolox equivalent/L, respectively [63,64]. The ratio of TOS to TAS comprises the oxidative stress index (OSI), which is used as an indicator for total oxidative stress [65].

### 2.6. DNA Damage Measurement

The protective effects of extenders containing inositol on DNA damage of spermatozoa were investigated by intracellular 8-hydroxydeoxyguanosine (8-OHdG) ELISA and comet assay.

#### 2.6.1. Measurement of 8-Hydroxydeoxyguanosine (8-OHdG) in Fish Sperm

8-Hydroxydeoxyguanosine (8-OHdG) is a significant sign of oxidant-induced DNA damage. Quantification of 8-OHdG was carried out using a fish ELISA kit (BT-LAB), following the manufacturer’s instructions.

#### 2.6.2. Comet Assay

Alkaline single-cell gel electrophoresis analysis (comet assay) was used to study the potential preventive effects of inositol on cryo-induced genotoxic DNA damage in fish sperm. The comet assay was carried out according to Singh et al. [53] with slight modifications as follows: approximately 2 × 10^4^ sperm cells were suspended in low-melting-point agarose (LMA) (75 µL of 1.0%) and stratified onto semi-frozen slides, previously covered with a thin layer of normal-melting-point agarose (1.0%). Another layer of 0.5% LMA was put over the second layer. The cells were dissolved for 2 h at 4 °C in a solution (100 mM EDTA, 2.5 M NaCl, 10% DMSO, 1% Triton X-100, 10 mM Tris, pH 10.0). Following dissolution, the slides were exposed to electrophoresis in buffers (0.3 M NaOH, 1 mM EDTA, pH 13.1) for 30 min. Then, the slides were neutralized with a Tris buffer (0.4 M Tris-HCl, pH 7.5). The slides were carefully dried at 25 °C in an incubator and marked with ethidium bromide (10 µg/mL in distilled water, 70 µL/slide). The slides were screened with the use of a fluorescence microscope imaging system (Leica DM1000, Solms, Germany). A hundred cells were randomly scored in each sample on a scale of 0–4 based on fluorescence beyond the nucleus. The scores were as follows: 0, no tail; 1, comet tail half the width of the nucleus; 2, comet tail equal to the width of the nucleus; 3, comet tail longer than the nucleus; and 4, comet twice the width of the nucleus. Scoring cells in this way has been shown to be as accurate and precise as using computerized image analysis [66].

### 2.7. Apoptotic Analysis by ELISA of Fish Sperm Cells

Analysis of apoptosis in sperm samples was performed according to the protocol of the commercially available kit (Cell Death Detection ELISA^PLUS^, Sigma-aldrich, Roche, Mannheim Germany). The assay is based on the quantitative double-antibody sandwich enzyme immunoassay principle and uses monoclonal antibodies directed against DNA and histones, respectively. An anti-DNA POD antibody binds to single- and double-stranded DNA. Therefore, the ELISA allows for the detection of mono- and oligonucleosomes from various species and can be applied to measure apoptotic cell death in many different cell systems.

### 2.8. Statistics

Minitab 17.0 was used for analysis. Normality and homogeneity of variance of the data were checked using the Kolmogorov–Simirnow test before analyses. The data that did not yield a normal distribution underwent logarithmic transformation. Percent motility and apoptotic cell data were arcsine transformed and analyzed with a one-way analysis of variance (ANOVA). The data generated were analyzed with the one-way ANOVA test, and the differences in the data of the groups were revealed by using the Tukey multiple comparison test. Linear or quadratic trends over the inositol level and sperm variables were determined using orthogonal polynomials [67] (SPSS V26.0). All mean values represent mean ± SE from triplicate. All data were represented as mean and standard error (mean ± SE).

## 3. Results

### 3.1. Evaluation of Sperm Parameters, Oxidant and Antioxidant Status, DNA Damage

In the fresh semen, spermatozoa motility rate, motility duration and spermatozoa concentration were 83.33 ± 1.67%, 112.44 ± 1.72 s and 12.11 ± 0.61 × 10^9^/mL, respectively. The motility rate and duration, apoptosis, DNA damage and total antioxidant and oxidant status of the spermatozoa were evaluated in the thawed semen from frozen Silurus triostegus with glucose extenders containing different doses of inositol. As shown in Table 1, Table 2, Table 3 and Table 4, semen parameters, DNA damage rates and oxidant and antioxidant status were significantly changed through the use of a glucose extender with inositol.

#### 3.1.1. Effect of Inositol on Post-Thaw Sperm Motility and Duration of Motility

Spermatozoa motility rate and motility duration are shown in Table 1. Increasing the levels of inositol significantly affected the spermatozoa motility rate and motility duration (*p* < 0.05). There were significant linear and quadratic trend levels of inositol for the spermatozoa motility rate and motility duration (*p* < 0.05), where the spermatozoa motility rate and motility duration increased with the inclusion of 40 mg and 20 mg levels of inositol, respectively.

#### 3.1.2. Effect of Inositol on Post-Thaw Antioxidant and Oxidant Status of Sperm

Levels of TAS, GSH, MDA, TOS and OSI were significantly affected by the inositol-supplemented extender (*p* < 0.0001). Significant linear and quadratic trends were found between the increasing levels of inositol and the levels of OSI (*p* < 0.05). Moreover, a linear trend was found between the increasing levels of the inositol and levels of TAS, GSH, MDA and TOS (*p* < 0.05).

#### 3.1.3. Changes in DNA Damage Parameters and Apoptosis in Post-Thaw Sperm with Frozen Glucose Diluent Supplemented with Inositol

As shown in Table 3, DNA damage in 8-OHdG and the comet assay were significantly affected by the inositol levels (*p* < 0.05). Significant linear and quadratic trends were found between the increasing levels of inositol and the level of DNA damage (8-OHdG and the comet assay) (*p* < 0.05).

As shown in Table 4, the percentage of apoptotic spermatozoa was significantly affected by the inositol levels (*p* < 0.05). Significant linear and quadratic trends were found between the increasing levels of inositol and the percentage of apoptotic spermatozoa (*p* < 0.05).

## 4. Discussion

Sperm cryopreservation is a method that preserves motility to a certain extent after thawing. The good results of sperm cryopreservation are dependent on sperm parameters and environmental factors such as the composition of the extender, the dilution rate and freezing and thawing procedures [30]. It is known that cryopreservation impairs sperm quality and reduces motility-related fertility [30,31]. Additionally, freezing causes a loss of membrane integrity, mitochondrial damage, metabolic and functional status changes, lipid peroxidation and increased cytoplasmic ROS and DNA damage in most cases [32,33,34,48,68].

In the studies in which buffalo (*Bubalus bubalis*) and red deer (*Cervus elaphus*) sperm was frozen, it was shown that including antioxidants in the semen extender had a positive effect on sperm motility [23,24]. Similarly, the sperm motility rate and motility duration of *Silurus triostegus* significantly improved with the increase in the inositol levels because the trends were linear and quadratic in this study. The addition of 40 mg of inositol to the sperm extender significantly boosted sperm motility and motility duration (Table 1). Functionally, in semen with oligoasthenoteratozoospermia, inositol acts directly on mitochondria by increasing membrane potential [32]. This may be due to the role of inositol in the osmoregulation of seminal fluid. Indeed, both hypo- and hyperosmotic environments have been found to increase sperm damage [25,26]. These morpho-functional data have recently been shown in studies, suggesting that sperm supplemented with high doses of inositol have significantly higher motility, possibly with increased cytosolic Ca^++^ and thus internal mitochondrial Ca^++^ [27].

The freeze–thaw process of sperm is related with oxidative stress, which gives rise to decreases in sperm function. Oxidative stress results from increased ROS production and decreased antioxidant levels that make sperm more vulnerable to lipid peroxidation [25,28,34,48]. The antioxidant potency of biological fluids can be assessed by measuring individual antioxidants or total antioxidant capacity. A positive correlation was found between seminal plasma and blood serum TAS levels [69]. Glutathione (GSH), which is found in semen and is involved in an antioxidant defense, comes from the seminal plasma. Glutathione has an important role against ROS and catalyzes the conversion of the first ROS, superoxide anion (O_2−_), to water (H_2_O_2_) [70]. The decrease in the sperm TOS and OSI levels after thawing may be due to the significant linear increase in the TAS and GSH levels in the extender caused by the increase in the extender inositol level. A significant effect was found between sperm oxidant and antioxidant levels and the extender inositol level in the present study. It was known that there was also close relationship between sperm oxidant and antioxidant levels and the extender antioxidant level. It has been reported that inositol addition prior to cryopreservation is likely to prevent from oxidative stress and remediate canine sperm quality after thawing [71]. A similar study with a different antioxidant has shown that the addition of curcumin to the freezing environment is preventive against human sperm parameters and sperm DNA and prevents oxidative damage caused by the freeze–thaw process [28]. The addition of antioxidants to the extender in cryopreservation may have improved semen quality after thawing [49]. The fact that inositol increases TAS level and improves sperm parameters is an indication that it is a powerful antioxidant. Although the underlying mechanisms are still largely unclear, MI has been among the antioxidants that can improve major sperm parameters [32,72]. It has been reported that this compound can make oxidative mechanisms more effective by increasing cytosolic and mitochondrial calcium levels, thus improving sperm mitochondrial function [32]. Recently, clear evidence has been presented that this potent antioxidant specifically acts by enhancing OXPHOS activity both at baseline and, to a greater extent, at capacitation in vitro [54]. In a study applying different concentrations of GSH (5, 7.5 and 10 mM), it was possible to observe high mitochondrial activity in refrigerated or thawed semen samples at a dose of 5 mM GSH [73]. The reducing stress effect of high doses of inositol on mitochondria causes an increase in reactive oxygen species and mitochondrial dysfunction, resulting in dysfunctions in ATP synthesis [74]. Thus, it can be stated that high doses of inositol do not cause a stress effect [73]. Low-intensity oxidative stress can be helpful to cells, whereas high levels can bring about degradation of nucleic acids, proteins, lipids and carbohydrates, ultimately leading to cell death [38,43,44].

Malondialdehyde is the final product of lipid peroxidation [28]. The level of lipid peroxidation determined by MDA was significantly reduced in semen after thawing with the addition of inositol to the semen extender. When free oxygen radicals rise, it leads to lipid peroxidation in sperm [40]. The lipid peroxidation product MDA causes DNA damage [39,40,41,42]. Inositol counteracts intracellular ROS, such as melatonin, which has antioxidant properties, thus reducing MDA formation and increasing its cryoprotective effects on spermatozoa, resulting in an increase in motility after thawing [28]. Park et al. [38] found that ROS production increased during the freezethaw process of semen. Lycopene added to the bovine sperm extenders reduced the production of reactive oxygen species and intracellular superoxide while a concurrent significant increase was observed in the concentration of MDA, the end product of lipid peroxidation, in both the seminal plasma and sperm cells of rooster [52,75].

The supposition that supplementation with a high dose of inositol to the extender decreased DNA damage was assessed by using 8-OHdG after freezing and thawing. Previous study results showed that acrylamide treatment resulted in a high plasma level of 8-OHdG, which was decreased when rats were treated with melatonin [76]. Similarly, Governini et al. [54] measured the level of 8-OHdG in treated and untreated sperm with MI (20 mg/mL) to evaluate the ability of MI to prevent DNA damage. A significant decrease in 8-OHdG in sperm has been demonstrated with MI. 8-OHdG, one of the early products of oxidative DNA damage, has been measured and it has been clearly shown that there is a significant decrease in this oxidative stress marker in the extenders supplemented with inositol [29]. The linear and quadratic relationship between oxidative DNA damage and inositol dose demonstrated in this study is consistent with previous studies, reporting a strong relationship between 8-OHdG levels and sperm functions [77,78].

Comet assay is considered one of the most sensitive and reliable tests [79]. In a study in which human, bull and mouse sperm DNA lesions were induced by DNAse I, whose effect in nuclei is dependent on the accessibility of chromatin and digests DNA without sequence specificity, the dose–effect relationship of the comet assay was different in the three species [36,80]. After the parameter curve reached a plateau at the lowest dose of the tested doses (1, 2, 10, 100, 1000 U/mL), no further increase in DNA migration was observed after 2 U/mL DNAse I concentration. In bull spermatozoa, a significant increase in comet parameters was obtained from the concentration of 100 U/mL DNAse I, which induced DNA damage in approximately 60% of spermatozoa. When the dose was increased, the mean level of damage increased and the damaged cells reached 100%. Mouse spermatozoa were found to be extremely resistant to endonuclease treatment, which did not show an increase in comet parameters, but at the highest dose, caused high levels of damage to 100% of spermatozoa [36]. With these results, it has been shown that the helical structure of chromatin provides protection against the effects of genotoxic factors in mammalian sperm. In the current study, due to the helical structure of catfish sperm chromatin, starting early, inositol may have protected against the DNA-degrading effect of fish sperm freezing factors [35,37].

These results of the comet assay were in agreement with the 8-OHdG results, since inositol prevents early stages (8-OHdG results) and the later stages of the sperm freez–thaw process (Comet assay results) [50]. In addition, the reduction of DNA damage may be due to the significant linear decrease in the MDA, TOS and OSI levels in sperm caused by the comparable increase in inositol level. These indicate that *Silurus triostegus* sperm can make good use of inositol to decrease the DNA damage.

Excessive and prolonged stress leads cells to apoptosis [81]. It is reported that inositol increases mitochondrial calcium levels and improves sperm mitochondrial function with its antioxidative effect and prevents apoptosis [32]. The high inositol level in sperm extender yielded the lower apoptotic sperm cell percentage. In addition trends were linear and quadratic. Apoptosis can occur within hours or even minutes [82,83]. ROS is also a known inducer of apoptosis in mature sperm [45,46]. It has been shown that exogenous ROS generation causes an increase in apoptosis in semen, and administration of antioxidants prevents the observed amount of DNA damage [84]. Mitochondria play an important role in ROS generation. Mitochondria are also involved in stress responses [47]. Hence, mitochondria play an important role in cell survival and can facilitate apoptosis when necessary [85]. It seems that if present at sufficiently high levels, inclusion of inositol to the extender is considerable for managing the levels of reactive oxygen, which may act as a trigger for apoptosis.

## 5. Conclusions

In *Silurus triostegus* sperm, high doses of inositol gave the highest percentage of the motility percentage, motility duration, TAS and GSH, but gave the lowest percentage of DNA damage, TOS, MDA levels and apoptotic cell rate. Hence, inositol can be used as an antioxidant additive in semen extenders for *Silurus triostegus* sperm, presenting better semen quality.

## Figures and Tables

**Table 1:** Spermatological parameters of *S. triostegus* after thawing (mean ± SE, *n* = 7).

Dose of Inositol (Groups)		Parameters	
		Motility Rate (%)	Motility Duration (s)
Control		50.00 ± 2.58 ^a^	55.00 ± 3.41 ^a^
5 mg		35.00 ± 2.14 ^b^	34.00 ± 2.49 ^b^
10 mg		29.00 ± 2.33 ^b^	29.8 ± 03.25 ^b^
20 mg		47.00 ± 2.13 ^a^	52.00 ± 3.55 ^a^
40 mg		51.00 ± 1.79 ^a^	56.80 ± 1.40 ^a^
Pr > F ^1^	ANOVA	0.00	0.00
	Linear trend	0.01	0.01
	Quatratic trend	0.32	0.03

Mean values within the same row with different superscripts are significantly different (*p* < 0.05). ^1^: Significance probability associated with the F-statistic.

**Table 2:** Oxidative stress status of *S. triostegus* sperm after thawing (mean ± SE, *n* = 7).

Dose of Inositol (Groups)		Parameters				
		TAS (mmol Trolox Equiv/L)	TOS (μmol H_2_O_2_ Equiv/L)	OSI (AU)	GSH (nmol/g)	MDA (nmol/g)
Control		1.11 ± 0.04 ^c^	16.03 ± 0.43 ^a^	1.24 ± 0.01 ^a^	27.53 ± 0.22 ^c^	22.17 ± 0.54 ^a^
5 mg		1.24 ± 0.01 ^bc^	15.37 ± 0.01 ^a^	1.28 ± 0.03 ^a^	30.67 ± 0.35 ^c^	18.45 ± 0.30 ^b^
10 mg		1.32 ± 0.03 ^b^	12.11 ± 0.25 ^b^	0.86 ± 0.07 ^b^	34.29 ± 0.13 ^b^	17.21 ± 0.31 ^b^
20 mg		1.80 ± 0.08 ^a^	9.47 ± 0.81 ^c^	0.52 ± 0.05 ^c^	37.84 ± 0.51 ^a^	8.54 ± 0.22 ^c^
40 mg		1.86 ± 0.02 ^a^	8.90 ± 0.81 ^c^	0.47 ± 0.01 ^c^	37.89 ± 0.37 ^a^	7.78 ± 0.17 ^c^
Pr > F ^1^	ANOVA	0.00	0.00	0.00	0.00	0.00
	Linear trend	0.00	0.00	0.00	0.00	0.00
	Quatratic trend	0.67	0.15	0.00	0.02	0.41

TAS, total antioxidant status; TOS, total oxidant status; OSI, oxidative stress index; GSH, glutathione; MDA, malondialdehyde; AU, arbitrary unit. Data are presented as mean ± SE of values. Mean values within the same row with different superscripts are significantly different (*p* < 0.05). ^1^: Significance probability associated with the F-statistic.

**Table 3:** DNA damage of *S. triostegus* after thawing using comet assay and 8-hydroxydeoxyguanosine (8-OHdG) (mean ± SE, *n* = 7).

Dose of Inositol (Groups)		Parameters	
		8-OHdG (ng/mL)	Comet (AU)
Control		140.00 ± 1.00 ^a^	37.52 ± 1.27 ^a^
5 mg		110.15 ± 0.21 ^c^	28.74 ± 0.47 ^b^
10 mg		115.07 ± 1.03 ^c^	24.03 ± 0.39 ^c^
20 mg		125.00 ± 1.00 ^b^	22.65 ± 0.21 ^c^
40 mg		73.01 ± 0.30 ^d^	10.55 ± 0.19 ^d^
Pr > F ^1^	ANOVA	0.00	0.00
	Linear trend	0.01	0.00
	Quatratic trend	0.00	0.00

8-OHdG, 8-hydroxydeoxyguanosine; Comet, comet assay; AU, arbitrary unit. Mean values within the same row with different superscripts are significantly different (*p* < 0.05). ^1^: Significance probability associated with the F-statistic.

**Table 4:** Analysis of *S. triostegus* apoptosis after thawing using ELISA (mean ± SE; *n* = 7).

Dose of Inositol (Groups)		Parameters
		Apoptozis (AR %)
Control		2.45 ± 0.04 a
5 mg		1.98 ± 0.03 ^b^
10 mg		1.84 ± 0.03 ^c^
20 mg		1.31 ± 0.03 ^d^
40 mg		1.22 ± 0.02 ^d^
Pr > F ^1^	ANOVA	0.00
	Linear trend	0.00
	Quatratic trend	0.00

AR, apoptotic rate. Mean values within the same row with different superscripts are significantly different (*p* < 0.05). ^1^: Significance probability associated with the F-statistic.

## Data Availability

The datasets used and/or analyzed during in this study are available from the corresponding author upon reasonable request.
